# Concomitant Paratubal Cyst and Isolated Fallopian Tube Torsion Masquerading as Acute Appendicitis in a Pregnant Female

**DOI:** 10.7759/cureus.46578

**Published:** 2023-10-06

**Authors:** Thomas G Mackay, Millie P Williams, Elena Kreimer, Francis Asomah

**Affiliations:** 1 School of Medicine and Dentistry, Griffith University, Gold Coast, AUS; 2 Department of Surgery, Mount Isa Hospital, Mount Isa, AUS; 3 Department of Obstetrics and Gynecology, Mount Isa Hospital, Mount Isa, AUS

**Keywords:** abdominal pain in pregnancy, diagnosis of acute appendicitis, diagnosis of appendicitis in pregnancy, isolated fallopian tube torsion, paratubal cyst

## Abstract

Abdominal pain in pregnant individuals presents diagnostic challenges, especially when appendicitis is suspected. We report a rare case of a 26-year-old pregnant female with recurrent right lower quadrant (RLQ) abdominal pain initially misdiagnosed as a urinary tract infection. Diagnostic uncertainty led to a magnetic resonance imaging (MRI) scan, which revealed a right adnexal cystic structure and a thickened tubular structure adjacent to the cecal pole, raising concerns of complicated appendicitis. Subsequent diagnostic laparoscopy revealed a right-sided fallopian tube paratubal cyst with 360-degree torsion and associated fallopian tube torsion without the involvement of the ovary. The cyst was successfully excised, and the patient subsequently delivered a healthy baby via emergency lower section caesarean section.

Abdominal pain during pregnancy has various causes. Diagnosing appendicitis during pregnancy is challenging due to anatomical and physiological changes. Ultrasound (US) is commonly used but has limited accuracy. Computed tomography (CT) is avoided due to radiation risks, while MRI is increasingly used and shows high diagnostic accuracy or aids in alternative diagnoses. Regardless of the diagnosis, the prompt recognition of intraabdominal pathology is crucial to prevent fetal morbidity.

This case highlights the challenges in the accurate diagnosis of abdominal pain during pregnancy and emphasizes the importance of considering alternative pathologies to prevent delays in treatment and complications. Clinicians should consider diagnostic laparoscopy for pregnant patients with equivocal investigations and lower abdominal pain. The differential diagnosis may include both common and rare causes such as concomitant paratubal cyst and isolated fallopian tube torsion (IFTT), emphasizing a high index of suspicion and collaboration with obstetric colleagues to ensure optimal care.

## Introduction

Abdominal pain during pregnancy presents a complex diagnostic challenge. Similar to non-pregnant females, abdominal pain in pregnant individuals can arise from various organs and pathological processes. However, one crucial diagnosis that requires diligent evaluation is appendicitis. Healthcare professionals frequently seek a general surgery consultation when confronted with abdominal pain during pregnancy, primarily to exclude the possibility of appendicitis. Recent reports indicate that appendicitis occurs in approximately one in 1500 pregnancies [1]. Although it commonly presents in the second and third trimesters, it can occur at any stage of pregnancy. The diagnostic process is further complicated by anatomical, physiological, and biochemical changes that occur during pregnancy, as well as safety concerns associated with conventional imaging techniques such as computed tomography (CT), which reduce the sensitivity of the non-pregnant females’ diagnostic assessment. Consequently, a more proactive surgical approach is often chosen, leading to a higher rate of negative appendectomies in pregnant females and the associated post-operative morbidity [2]. Obstetricians and surgeons must maintain a high level of suspicion for acute appendicitis, as untreated cases can result in adverse outcomes such as preterm labor, premature delivery, fetal distress, growth restriction, and fetal loss [3]. However, it is crucial to avoid overlooking the wide range of potential causes of abdominal pain in pregnancy, including rarer non-general surgical pathologies that should be considered.

## Case presentation

**Figure 1:**
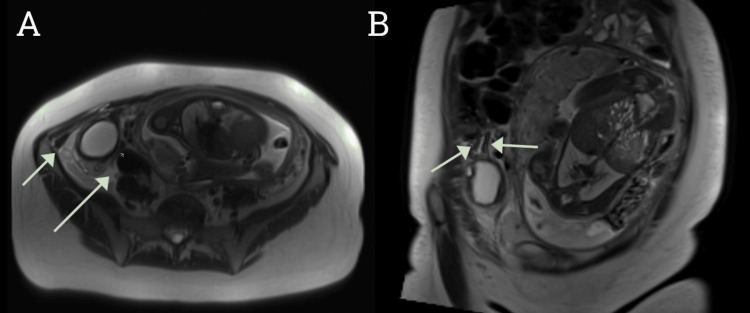
(A) Axial and (B) coronal MRI abdominal images demonstrating inflammatory change adjacent to a cystic structure near the right ovary and thickened tubular structure adjacent to the cecal pole. The arrows show areas demonstrating inflammatory changes. MRI: magnetic resonance imaging

A 26-year-old Caucasian female with a background history of being 33 weeks and four days pregnant and a laparoscopic sleeve gastrectomy presented to the emergency department of our remote hospital with acute on recurrent right lower quadrant (RLQ) abdominal pain for six days. Her pregnancy was also complicated by contracting cytomegalovirus, with close monitoring enacted to ensure appropriate fetal growth. The patient presented to the pregnancy assessment center six days prior to the presentation and was diagnosed with a urinary tract infection based on a contaminated urine sample (leukocytes, 200 × 10^6^/L; erythrocytes, <10 × 10^6^/L; epithelial cells, >50 × 10^6^/L) and abdominal pain. She was commenced on oral cefalexin and discharged home. Two days later, she re-presented with an episode of severe RLQ abdominal pain with associated nausea and vomiting. She was given intravenous ceftriaxone and, due to carer responsibilities, discharged home. She once again re-presented on day 5 of the initial onset of symptoms. On arrival, she was found to have tachycardia of 120 beats/minute and otherwise normal observations. Her abdomen demonstrated laparoscopic scars, with a gravid uterus palpable above the umbilicus. She demonstrated tenderness and percussion tenderness in the RLQ of the abdomen, particularly over a palpable mass. She underwent an ultrasound (US) scan of her pelvis, which reported a right adnexal cystic structure measuring 55 × 52 × 37 mm, presumed to be a simple ovarian cyst. Despite continued antibiotics and opioid analgesia, blood investigations demonstrated a C-reactive protein (CRP) of 114 g/dL with a normal white cell count. A general surgery consult was arranged as complicated appendicitis was proposed as a differential diagnosis by the treating gynecology team. Her persistent abdominal pain and clinical suspicion necessitated a magnetic resonance imaging (MRI) scan of her abdomen (Figure 1). This scan again noted a right adnexal cystic structure, favoring a simple right ovarian cyst; however, this scan also noted a thickened tubular structure adjacent to both the cecal pole and the right adnexal mass.

**Figure 2:**
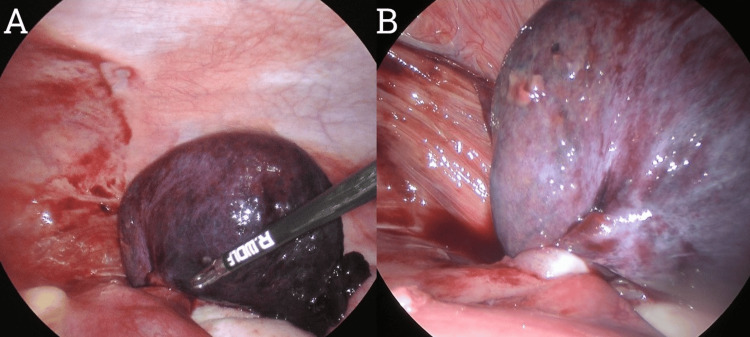
Laparoscopic images demonstrating (A) the concomitant torted right paratubal cyst and isolated fallopian tube and (B) a close-up of the 360-degree torsion arising from the intact right fallopian tube.

**Figure 3:**
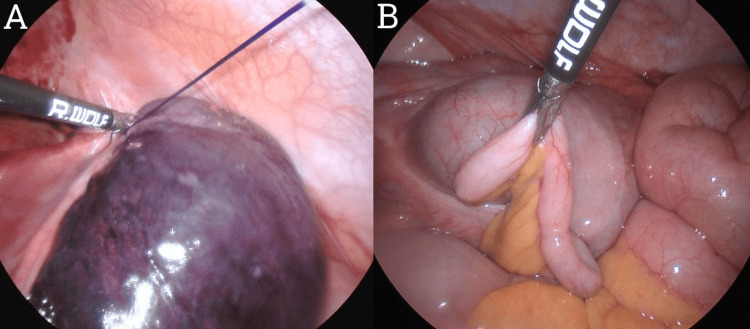
Laparoscopic images demonstrating (A) the application of an ENDOLOOP® Ligature to the pedicle of the torted right paratubal cyst and isolated fallopian tube and (B) the normal-appearing appendix.

**Figure 4:**
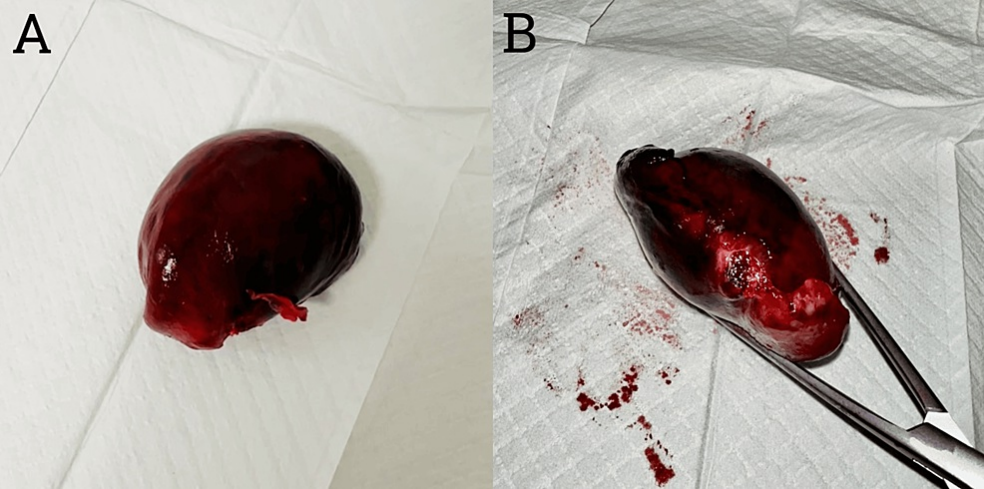
Clinical images of the gross specimen (A) on the anti-pedicle side and (B) the pedicle side.

Considering the diagnostic uncertainty and possibility of appendicitis, a diagnostic laparoscopy was performed the next day. Intraoperatively, the patient was found to have a right-sided fallopian tube paratubal cyst (Figure 2A) with evidence of 360-degree torsion (Figure 2B). The cyst was hemorrhagic and necrotic with adjacent free fluid in the RLQ and pelvis. The necrotic cyst was looped with an ENDOLOOP® Ligature (Ethicon, Inc., Cincinnati, OH) (Figure 3A) before the cyst was divided from its pedicle with laparoscopic monopolar diathermy and scissors. The right fallopian tube was grossly preserved. The right ovary was normal in gross appearance without evidence of cysts, and the appendix was normal in gross appearance and left in situ (Figure 3B). The histology of the operative specimen (Figure 4) demonstrated an 80 × 60 × 40 mm torted cystic structure consisting partially of the fallopian tube but predominantly a large paratubal cyst without evidence of dysplasia or malignancy. The patient was readmitted to the hospital on the seventh post-operative day for decreased fetal movements, and she underwent an emergency lower section caesarean section without complication. At the time of submission, the patient had no other post-operative complications. Thus, we present a rare case of paratubal cyst and isolated fallopian tube torsion (IFTT) in pregnancy masquerading as complicated acute appendicitis.

## Discussion

The diagnosis of appendicitis poses significant challenges during pregnancy due to various factors. The presence of a gravid uterus leads to the displacement of intraabdominal contents, including the cecal pole and appendix, thereby diminishing the reliability of abdominal examination. Moreover, physiological changes such as elevated heart rate, circulating white blood cell count, and CRP levels further obscure the diagnosis. In our practice, ultrasound (US) scan is the initial imaging modality employed for assessing acute appendicitis in pregnant females due to its accessibility and cost-effectiveness. However, its sensitivity and specificity in this context are limited. Computed tomography (CT) scans are generally avoided due to potential harm from ionizing radiation exposure to the fetus. Conversely, the utilization of magnetic resonance imaging (MRI) to diagnose appendicitis during pregnancy has increased in recent times. MRI has been reported to exhibit a sensitivity ranging from 90% to 100% and a specificity ranging from 87% to 98% for diagnosing acute appendicitis in pregnancy and thus is an important investigation [4]. While our local protocol favors the use of 1.5 T MRI to minimize fetal heating concerns, the American College of Radiology supports the safe use of MRI in all trimesters [5]. The prompt diagnosis of appendicitis is crucial during pregnancy to mitigate significant fetal morbidity and, in severe cases, fetal loss.

Abdominal pain during pregnancy can have various causes, some of which can also result in fetal morbidity. Paratubal cysts, which are typically benign fluid-filled sacs lined by ciliated cuboidal cells originating from Mullerian or Wolffian remnants [6], are often incidentally discovered during imaging or laparoscopic procedures and are generally asymptomatic [7]. However, these cysts can become complicated by torsion, hemorrhage, or infection. Paratubal cysts are frequently misidentified as ovarian masses and can be a common cause of tubal torsion [8]. Isolated fallopian tube torsion (IFTT), a rare condition characterized by the torsion of the fallopian tube without associated ovarian torsion, can occur during pregnancy [9]. Unfortunately, there are no pathognomonic features for the diagnosis, resulting in frequent misdiagnosis or delayed diagnosis [10]. While the diagnosis in the current case did not involve appendicitis, the morbidity associated with an untreated necrotic and hemorrhaging cystic structure is expected to be significant, and diagnostic laparoscopy was certainly warranted.

## Conclusions

In summary, general surgeons should have a low threshold to consider diagnostic laparoscopy in patients with equivocal blood and imaging investigations who have lower abdominal pain during pregnancy. The differential diagnosis is wide and varied, and many pathologies may masquerade as appendicitis. While rare, paratubal cysts as a cause of IFTT should be considered in the differential diagnosis of abdominal pain in pregnancy, especially when a presumed ovarian cyst is observed on imaging. A high index of suspicion and collaboration with obstetric colleagues is necessary to preserve future fertility and avoid morbidity of the pregnant mother and fetus.

## References

[1] Mazze RI, Kallen B (1991). Appendectomy during pregnancy: a Swedish registry study of 778 cases. Obstet Gynecol.

[2] Walsh CA, Tang T, Walsh SR (2008). Laparoscopic versus open appendicectomy in pregnancy: a systematic review. Int J Surg.

[3] Winter NN, Guest GD, Bozin M (2017). Laparoscopic or open appendicectomy for suspected appendicitis in pregnancy and evaluation of foetal outcome in Australia. ANZ J Surg.

[4] Dewhurst C, Beddy P, Pedrosa I (2013). MRI evaluation of acute appendicitis in pregnancy. J Magn Reson Imaging.

[5] ACR Committee on MR Safety (2020). American College of Radiology: ACR. ACR manual on MR safety.

[6] Atal R (2016). Torsion of paraovarian cyst resulting in secondary torsion of ovary and fallopian tube. Int J Obstet Gynaecol Res.

[7] Samaha M, Woodruff JD (1985). Paratubal cysts: frequency, histogenesis, and associated clinical features. Obstet Gynecol.

[8] Alpendre F, Pedrosa I, Silva R, Batista S, Tapadinhas P (2020). Giant paratubal cyst presenting as adnexal torsion: a case report. Case Rep Womens Health.

[9] Meyer R, Meller N, Mohr-Sasson A (2022). Clinical features of isolated fallopian tube torsion: evidence from a large series. Hum Fertil (Camb).

[10] Baracy MG Jr, Hu J, Ouillette H, Aslam MF (2021). Diagnostic dilemma of isolated fallopian tube torsion. BMJ Case Rep.

